# Surgical Consideration of Peripheral Nerve Injuries

**Published:** 1919-01

**Authors:** 


**Surgical Consideration of Peripheral Nerve Injuries.** By BYRON STOOKEY. *Surgery, Gynecology and Obstetrics*, October, 1918.

In this paper the author deals with the indications for surgical interference, and the technic of peripheral nerve surgery.

It is a fundamental principle of nerve surgery that all operations should be done in a sterile field and in the absence of the possibility of infection. Hence, secondary suture is the rule. Recrudescence of infection in an apparently healed wound must always be feared; therefore sufficient time must elapse to insure a clean, non-infected field.

The aim of surgical interference is to facilitate the normal process of repair by removing all causes hindering the downgrowth of the neuraxes, and to facilitate their growth either through normal paths, i. e. by end-to-end suture, or down newly furnished paths, be they nerve transplants, or artificial conducting paths.

It is generally impossible to determine whether an injured nerve will recover without operation, except by waiting long enough to allow for evidences of nerve degeneration to manifest themselves. It is most important, therefore, to study carefully the progressive changes in the pathological signs.

So long as there are progressive signs of nerve regeneration, no surgical interference should take place. The earliest and most reliable signs of regeneration are : formication, shrinkage of areas of anesthesia, return of deep sensibility, return of muscle tone, and muscle sensibility. Usually in from 3 to 4 months some signs of nerve regeneration should be evident. By waiting so long no great time is lost, as usually during the greater part of this period the wound has been infected.

From a purely surgical standpoint, the earlier the operation is undertaken, the less difficult it is, and the better are its results.

Early exploratory operations are sometimes advisable, since, even with the most careful examination, it is impossible to distinguish lesions with anatomical loss of nerve substance from those with interruption of conductivity and no concomitant loss of anatomical continuity.

A general anesthetic should always be resorted to.

Contractures should be corrected, as far as possible, before operation.

An absolutely dry field during operation is most necessary. Bits of torn muscle held over the nerve ends, or simply gentle and prolonged digital pressure, are advantageous for checking hemorrhage. The use of the tourniquet is to be avoided; each bleeding point should be dealt with individually.

When the nerve is imbedded in much scar and callus, it will be found safer to dissect from sound and normal tissue into scar.

When the nerve is freed from the immediate scar, it can be freed and liberated in its contiguous parts by gentle traction. This is best done before excising the intervening scar. Squeezing the nerve ends to make traction must be carefully avoided.

It is good procedure to cut only partially through the scar, using it as a means of fixation for the nerve ends. It is easier to suture with the ends fixed, and, by so suturing, there is less tendency to axial rotation and greater accuracy in maintaining the internal topography of the nerve. The more accurate the union, the more complete and the earlier the return of function.

Small, smooth, half-curved needles, with either fine silk or plain catgut, should be used.

The suture should include as little of the nerve as possible. The nerve ends should be brought gently into alignment without pressure or tension.

It is often impossible even at operation to determine whether or not nerve axes have reached the distal stump. If the axes are new and have not as yet reformed the motor end plates, there will be no electrical response, even though they have reached the distal stump. Correct judgment can only be gained by wide experience, and by checking observations by microscopical examinations of the excised tissue.

The 3 main types of operation are : nerve liberation, suture, either complete or partial, and nerve grafting.

Nerve liberation is successful only when the nerve trunk has been completely freed, and when there is no scar within the nerve. Otherwise excision and suture must be resorted to.

In such cases where only a very slight interstitial scar exists, conservative measures may be attempted; by injecting salt solution under pressure, into the nerve, adhesions may be broken up, and new channels opened for the neuraxes.

Direct nerve suture should only be done when the nerve ends can be approximated without tension. Gentle traction may be used, but too strenuous a pull must be carefully avoided. By means of altering the position of the limb, a few centimeters may be gained in some instances. When excising *scar* within the nerve trunk, the excision should be as liberal as necessary, with no regard to the amount of *scar* removed.

Partial nerve suture is frequent in injuries of the larger nerves. The aim is to remove all interstitial scar without section of the normal nerve funiculi.

When the loss of nerve substance is considerable, grafting must be resorted to. The nerves used are the radial, just below the elbow, and the musculocutaneous of the arm and leg, either as single or multiple grafts.

When possible, the nerve should be very loosely surrounded by a pedicle of fat selected with some consideration as to blood supply. The nerve trunk may also be transposed among muscle bellies or imbedded in some adjacent muscle.

In a few particular injuries it may be deemed expedient to do tendon transplantation rather than some form of nerve bridging.

Injuries to blood vessels, including thrombosis and aneurysms, occur frequently as complications in operations for the repair of peripheral nerves. Occasionally they may not be diagnosed in time and may lead one into extreme operative difficulties.

In cases in which there is much pain, operation should be resorted to, even if there is some evidence of progressive nerve regeneration. In cases of causalgia immediate relief has been obtained sometimes after freeing the nerve from the scar, the callus, or from around bony exostoses. When the cause lies within the nerve, injections of 60 per cent. alcohol have been recommended.

